# Is There a Link between Zinc Intake and Status with Plasma Fatty Acid Profile and Desaturase Activities in Dyslipidemic Subjects?

**DOI:** 10.3390/nu12010093

**Published:** 2019-12-28

**Authors:** Marija Knez, Ana Pantovic, Milica Zekovic, Zoran Pavlovic, Maria Glibetic, Manja Zec

**Affiliations:** 1 Centre of Research Excellence in Nutrition and Metabolism, Institute for Medical Research, University of Belgrade, 11000 Belgrade, Serbia; jelenkovicana5@gmail.com (A.P.); zekovicmilica@gmail.com (M.Z.); mglibetic@gmail.com (M.G.); manjazecimr@gmail.com (M.Z.); 2 Institute for Public Health Pozarevac, Jovana Serbanovica 14, 12000 Pozarevac, Serbia; zpavlovicpo@yahoo.com

**Keywords:** Zn deficiency, Zn status, fatty acids, dyslipidemia, desaturases, dietary Zn intake

## Abstract

The prevalence of obesity and dyslipidemia has increased worldwide. The role of trace elements in the pathogenesis of these conditions is not well understood. This study examines the relationship between dietary zinc (Zn) intake and plasma concentrations of Zn, copper (Cu) and iron (Fe) with lipid profile indicators, fatty acid composition in plasma phospholipids and desaturase enzyme activities in a dyslipidemic population. The role of the newly proposed biomarker of Zn status, the linoleic:dihomo-gama-linolenic acid (LA:DGLA) ratio, in predicting Zn status of dyslipidemic subjects has been explored. The study included 27 dyslipidemic adults, 39–72 years old. Trace elements were determined using atomic absorption spectrometry and fatty acid composition by a liquid gas chromatography. Desaturase activities were calculated from product-precursor fatty acid ratios. Dietary data were obtained using 24 h recall questionnaires. Insufficient dietary intake of Zn, low plasma Zn concentrations and an altered Cu:Zn ratio is related to modified fatty acid profile in subjects with dyslipidemia. Plasma Zn status was associated with obesity. There was no correlation between dietary Zn intake and plasma Zn status. The LA:DGLA ratio was inversely linked to dietary Zn intake. Cu, in addition to Zn, may directly or indirectly, affect the activity of desaturase enzymes.

## 1. Introduction

The number of people affected by obesity and dyslipidemia has increased significantly during the last two decades, largely attributed to the changes in dietary and lifestyle habits. Sixty-five percent of the world’s population live in countries where overweight and obesity harm more people than malnutrition [1]. According to the World Health organization (WHO) the worldwide obesity rates have nearly tripled since 1975 [1]. In addition, the incidence of obesity related diseases (i.e., hypertension, dyslipidemia, metabolic syndrome) also increases [1,2]. Dyslipidemia is described as the presence of non-optimal blood lipids levels. People with dyslipidemia have either increased concentrations of triglycerides or low-density lipoprotein cholesterol (LDL-C), or decreased levels of high-density lipoprotein cholesterol (HDL-C) [3,4]. Raised cholesterol levels cause on average 2.6 million deaths and 29.7 million disability adjusted life years (DALYS) [1]. Dyslipidemia is one of the major contributors to atherosclerotic and ischemic heart disease [1,5,6]. In 2008, the prevalence of raised blood lipid levels among adults globally was 39% [1] and WHO identified that the occurrence was the highest in the region of Europe (54% for both sexes). Successful treatment of dyslipidemia significantly reduces morbidity and mortality from cardiovascular diseases [7]. Basically 10% reduction in serum lipid levels has been reported to result in a 50% reduction in heart disease within 5 years [1].

Trace elements have crucial roles in metabolism, growth, immunological and neurological functions [8,9,10]. Zinc (Zn) is required for an adequate activity of more than 300 enzymes involved in protein synthesis, fatty acid metabolism, reproduction and oxidative clearance [11]. Iron (Fe) is needed for proper oxygen transport, while copper (Cu) is important for the oxido-reduction and detoxification and is involved in growth, development of cellular elements of arterial walls, lipoprotein metabolism and immune function [9,12,13]. The concentrations of serum/plasma minerals Zn, Cu and Fe are associated with the development of chronic diseases [10,14,15]. Deficiency in microelements leads to an increase in fat deposition and obesity [16]. Hypoferremia is often seen in people suffering from hypertension and plasma Zn concentrations have been directly correlated with total cholesterol and LDL-C levels [15,17]. Zn, Cu and Fe plasma levels are related to the extent of myocardial damage [18]. Epidemiological studies show a direct correlation between low plasma Zn and Fe concentrations and increased risk of cardiovascular diseases [19]. Elevated plasma levels of Cu were reported in patients with coronary artery disease [20]. Although deficiencies of trace elements may alter metabolism of lipids and lipoproteins, the mechanism of their action is not yet completely understood. The fatty acid (FA) composition in serum phospholipids remains a reliable biomarker of long-term fat intake [21] and it can predict the development of metabolic and cardiovascular diseases [22]. It shows the endogenous FA metabolism, regulated by desaturase enzymes (Δ5, Δ6 and Δ9). Desaturase enzymes convert dietary FAs to long-chain omega 3 and omega 6-polyunsaturated fatty acids (PUFAs), the latter with demonstrated role in the prevention of dyslipidemia and metabolic syndrome [23,24]. Serum FA composition also predicts long-term development of dyslipidemia and metabolic syndrome [25,26,27]. Furthermore, alterations in desaturase activities were seen in subjects who are obese and insulin resistant [28,29]. Trace elements have a role in FA metabolism. Zn is a cofactor for desaturases and elongases in endogenous FA synthesis [30,31,32,33,34,35], so the alterations in plasma levels of Zn possibly influence the activities of these enzymes and consequently the regulation of FA metabolism. Serum concentrations of Zn and Fe correlated with serum PUFA concentrations and activities of certain desaturases [36,37,38]. However, the role of micronutrients in dyslipidemia warrants further research. Linoleic/dihomo-gama-linolenic acid (LA:DGLA) ratio is linked with dietary Zn intakes and this ratio has been proposed as a novel biomarker of Zn status in both animals and humans [32,33,35,39]. The predictive potential of this ratio has not been investigated in dyslipidemic subjects so far. The primary aim of this study was to examine the relationship between plasma concentrations of Zn, Cu, and Fe with long-chain individual FAs, FAs belonging to de novo lipogenesis pathway and estimated desaturase activities in a group of dyslipidemic adults. The relationship of the microelements with anthropometric and lipid profile parameters was assessed. The LA:DGLA ratio, an emerging biomarker of Zn status, has been additionally examined for potential correlations with clinical parameters. Finally, the contribution of particular food groups to the relationships between trace elements and fat metabolism was evaluated.

## 2. Materials and Methods

### 2.1. Study Population

This cross-sectional study is the part of a larger nutritional intervention study registered as NCT03227497 (ClinicalTrials.gov). Eligible subjects were non-smoking adults at moderate cardiovascular risk, the latter defined as the presence of at least one of the following: increased body mass index (BMI = 25–30 kg/m^2^), central obesity (waist circumference ≥ 80 cm for women and ≥94 cm for men), and high normal blood pressure (systolic/diastolic blood pressure (SBP/DBP) ˃ 120/80, ≤139/89 mm Hg). Dyslipidemia was defined according to the guideline of the US National Cholesterol Education Program Adult Treatment Panel III (NCEP-ATP III) [7,40]. The exclusion criteria were the presence of chronic disease, self-reported allergy to any type of nuts, pregnancy, lactation, blood donation 16 weeks before the start of the study and a parallel participation in another clinical trial.

Recruitment of the subjects was performed via newspaper advertisement or word of mouth. For the purposes of the current sub-study, we selected subjects with disturbed levels of serum lipids, as seen in either: elevated serum total cholesterol (≥5.2 mmol/L), and/or elevated low-density lipoprotein cholesterol (LDL-C ≥ 3.4 mmol/L) and/or elevated serum triacylglycerides (TG ≥ 1.7 mmol/L) and/or decreased high-density lipoprotein cholesterol (HDL-C ≤ 1.04 mmol/L and 1.29 for men and women, respectively). Finally, we were left with 27 dyslipidemic subjects of both genders, who were examined in the current study to assess the relationship between micronutrient intake and status with fat metabolism.

### 2.2. Ethical Considerations

All subjects went through the informed consent process, both verbal and written. The study protocol was approved by the Institute for Medical Research, University of Belgrade, Belgrade, Serbia, Ethics Committee Approval, No: EO120/2017. The study was conducted in agreement with the ethical guidelines on biomedical research on human subjects of The Code of Ethics of the World Medical Association’s Declaration of Helsinki (1964) and its further amendments [41]. This study forms a part of the randomized clinical trial registered as NCT03227497 (ClinicalTrials.gov).

### 2.3. Blood Samples Collection and Analysis

Blood samples were collected after an overnight fast (>10 h fasting) between 7–8 a.m. by venepuncture of the antecubital vein. Tubes with ethylenediaminetetraacetic acid as anticoagulant were used, and all of them were free of trace elements. Lipid status and concentration of glucose, along with other routine biochemical parameters were determined from serum samples on the same day they were collected. For this purpose, a clinical chemistry analyzer Cobas c111 was used (Roche Diagnostics, Basel, Switzerland) and Roche Diagnostics’ kits according to the manufacturer’s instructions.

### 2.4. Anthropometric Measurements

Anthropometric variables, height and weight, were measured to the nearest 0.1 cm and 0.1 kg, respectively. The weight and percent of fat mass in the body composition of participants were measured using a TANITA UM072 balance (TANITA Health Equipment H.K. LTD, Hong Kong, China). The BMI was calculated as the ratio of weight (kg) to height squared (m^2^). BMI was used to assess the prevalence of overweight (25–29.9 kg/m^2^) and obesity (≥30 kg/m^2^) according to WHO criteria [1].

### 2.5. Assessment of Dietary Zn Intake

Dietary intake assessment was based on two 24 h recalls collected for non-consecutive days, one working day and one weekend day, to capture the intra-individual food consumption variability. Nutritional evaluation was performed during pre-scheduled participants’ visit to the study center within in-depth interviews conducted by trained researchers. Recalls were administered according to multiple-pass approach with standardized protocol, interview structure, and probing questions. To facilitate the evaluation of food servings and improve accuracy of reporting, participants were prompted with validated two-dimensional portion size estimation aid i.e., booklet comprising photograph-series illustrating a range of reference portions for 135 items (both simple foods and recipes) commonly consumed in the Balkan region [42]. In conjunction with the photographic atlas, household measures, package/labelling data, the standard serving size information and natural units were used to quantify the amount of food consumed.

Dietary questionnaires were coded and analyzed using Diet Assess & Plan, an advanced nutritional software tool, evaluated by European Food Safety Authority (EFSA) and successfully used in various national/regional surveys and international projects [43,44]. The consumption data was converted to nutrient intake estimates according to the Serbian Food Composition Database, compiled and managed in accordance with the international guidelines and embedded in the European Food Information Resource AISBL (EuroFIR AISBL) platform [45]. Nutrient intake calculation was performed by multiplying the estimated gram weight of the consumed food with the nutrient contents per gram from the food composition table. The average daily intakes of energy, macronutrients, trace elements, FAs, and the main food groups were derived from a mean of two administrations of the questionnaire.

### 2.6. Fatty Acid Determination and Estimation of Desaturase Activity

Fatty acid concentrations were determined by gas chromatography (GC). By use of chloroform-methanol mixture (2:1), total plasma lipids were extracted according to method of Folch et al. [46]. To prevent lipid peroxidation, 2,6-di-tert-butyl-4-methylphenol (10 mg/100 mL) were added to the mixture of solvents. Further on, phospholipids were separated from total lipids on silica thin-layer chromatography plate, on a mixture of petroleum ether, diethyl ether, and glacial acetic acid (87:12:1) used as a solvent system. Following the previously described procedure [47] with slight modifications [48] FA methyl-esters were further obtained by direct trans-methylation. Obtained FA methyl-esters were analyzed by gas chromatography on Shimadzu chromatograph GC 2014, Tokyo, Japan equipped with a flame ionization detector and a Rtx 2330 fused silica gel capillary column (60 m × 0.25 mm id × 0.2 μm film thickness) (Restek Co., Bellefonte, PA, USA) according to method by Veselinovic et al. [48]. For the purposes of current study oleic acid (18:1 *n*-9), alpha-linolenic acid (LNA, 18:3 *n*-3), linoleic acid (LA, 18:2 *n*-6), dihomo-γ-linolenic acid (DGLA, 20:3 *n*-6), arachidonic acid (ARA, 20:4 *n*-6), adrenic acid (AA, 22:4 *n*-6), eicosapentaenoic acid (EPA, 20:5 *n*-3), docosapentaenoic acid (DPA, 22:5 *n*-3), and docosahexaenoic acid (DHA, 22:6 *n*-3) were identified. The peak retention times were compared with certified calibration mixtures (PUFA-2, Supelco, Bellefonte, PA, USA, and 37 FAMEs mix, Sigma Chemical Co., St. Louis, MO, USA). The amounts of individual FA are presented as the relative percentage of the total (100%) FA pool. The desaturase activities were estimated as precursor-to-product ratios of individual FAs as follows: the ratio AA/DGLA (20:4 *n*-6/20:3 *n*-6) for delta 5 desaturase, GLA/AA (20:3 *n*-6/18:2 *n*-6) for delta 6 desaturase and 16:1 *n*-7/16:0 ratio for delta 9 desaturase [49].

### 2.7. Mineral Analysis

The analysis of plasma mineral concentrations was conducted using flame atomic absorption spectrometry on a Varian SpectrAA-10 (Varian, Inc., Mulgrave, Victoria, Australia) according to the method described by Jian Xin [50]. The reference range used for plasma Zn concentration was 0.7–1.5 mg/L; 0.5–1.5 mg/L was used for Fe and 0.7–1.4 mg/L for Cu [51,52,53]. To verify the accuracy of the method, two control serums with certified concentrations of zinc, cooper and iron, (ClinChek-Control, Recipe Chemical + Instruments GmbH; catalogue number 8882) were analyzed. Method performance was monitored by analysis of the same control serums within each of the series. The obtained results agreed with the certified values.

### 2.8. Statistical Analysis

The normality of the data was assessed with the Shapiro–Wilk test. Normally distributed data are presented as mean (standard deviation) (SD) and non-normally scattered as median [interquartile range]. The variability in the baseline parameters was determined depending on the plasma Zn status, stratified according to Zn tertiles. The total number of participants when divided according to tertiles was 23.

The difference between the tertiles in the selected variables was explored with one-way analysis of variance test for normally distributed data, and with Kruskal Wallis test for non-normally distributed data. To explore potential relationships between the study outcomes, the correlation analyses were employed. For normally distributed data, Pearson’s correlation tests were used, while for non-normally distributed data the corresponding non-parametric-Spearman’s correlation test was employed. To explore potential confounding effects, partial correlation tests were used, with addition of the following variables: age, gender, plasma Zn status. All analysis was performed in SPSS version 23.0 (BM Corp., Armonk, NY, USA), with a significance threshold set at 0.05. Graphical presentations were generated in RStudio (2019) (Version 1.2.1335, Boston, MA, USA) [54].

## 3. Results

### 3.1. Baseline Characteristics of Study Participants

Table 1 illustrates the baseline characteristics of study participants based on the tertiles of plasma Zn concentrations. The mean age of the participants was 56 with values ranging from 39–72. Forty-eight percent of this study population was overweight and 22% obese. Values for BMI fluctuated from 18.4 to 39.6, with a mean value of 28.2 kg/m^2^. Fifty-two percent of people had a waist:hip ratio above 0.86. The percentage of study subjects with high total cholesterol and high LDL-C was 88%, while 30% of the complete sample was with low levels of HDL-C. Increased level of TG, in addition to high LDL-C and low HDL-C, was seen in 18.5% of participants. The mean fasting blood glucose (FPG) was 5.17nmol/L, with 25% of people with glucose levels above the reference value of 5.84nmol/L. Fifty-seven percent of subjects had elevated blood pressure (reference range 130/80).

The average plasma Zn concentration was 0.75 ± 0.1 mg/L with 26% of participants having plasma Zn concentrations below 0.7 mg/L. More than 50% of study subjects were with plasma Zn levels below 0.75 mg/L, with the highest concentration of 1.03 mg/L being measured. The average Cu:Zn ratio was 1.3. The levels of Cu and Fe were within the recommended reference ranges for healthy people. The percentage of individual FAs and desaturase enzyme activities are presented in Table 1. The plasma levels of Zn increased with higher weight and BMI. No differences between the groups were seen for plasma concentrations of either Fe or Cu. There was a statistically significant difference in the Cu:Zn ratio between the tertile groups, with the highest ratio observed in the 1st Zn tertile. No statistically relevant relations were shown between the groups for any of the lipid profile indicators. Concentrations of the individual FAs did not differ between the Zn formed groups (*p* > 0.05).

### 3.2. Associations between Plasma Zn, Fe, and Cu Status with Anthropometrical and Biochemical Measures

There was a direct correlation between Zn and BMI, and a significant inverse relation between the Cu:Zn ratio and BMI. The relationships remained in the age and gender-adjusted model: Zn correlated directly (r = 0.536, *p* = 0.02), while Cu:Zn ratio inversely (r = −0.488, *p* = 0.02) with BMI. There was an inverse relationship between the Cu:Zn ratio with Cho/HDL, LDL/HDL and non-HDL/HDL (Table 2). Fe and Cu were not correlated with any of the biochemical parameters measured in this study (Table 2).

No statistically significant correlations were observed between the plasma concentrations of different minerals (*p* > 0.05). There is no statistically relevant association between the Fe status and anthropometry parameters except for the inverse correlation with the percentage of body fat (Table 2).

### 3.3. The Link between the Plasma Zn, Cu, and Fe Status with Total Phospholipid FA Composition and Desaturase Enzyme Activities

There were no significant correlations between the plasma Zn and any of the FAs measured in this study. Cu was directly related to oleic acid (18:1 *n*-9) and alpha-linolenic acid (ALA, C18:3 *n*-3). Plasma Fe correlated directly with oleic acid, while Cu:Zn ratio was directly associated with alpha-linolenic acid (Table 3).

No statistically significant relationships were seen among the plasma status of either Zn or Fe with any of the desaturases. There was an inverse correlation trend between the LA:DGLA and Zn and Fe status and a direct relationship between the Cu and LA:DGLA ratio, without reaching statistical significance. 

### 3.4. The Link between the Status of the Individual FAs with Certain Components of Dyslipidemic Status (Anthropometrical and Biochemical Parameters)

The linoleic acid correlated inversely, while arachidonic acid was directly linked to BMI (Table 4). No other significant links between the status of individual FAs and anthropometry indicators were observed. Similarly, no correlations were seen between the biochemistry related parameters and FAs, except for the direct correlations of oleic acid (C18:1 *n*-9) with total cholesterol and HDL-C (Table 4), and for the direct relationship between dihomo-gamma-linolenic acid (DGLA; C20:3 *n*6) and TGs.

### 3.5. Correlations between LA:DGLA Ratio and Estimated Desaturase Activities with FAs, Anthropometrical, and Biochemical Indices

The LA:DGLA ratio was inversely linked to BMI (Table 5). Predicted activities of Δ5 and Δ9 desaturases were not linked to any anthropometry measure, while Δ6 desaturases was directly related to BMI. LA:DGLA was inversely associated with TG, Cho/HDL ratio and non-HDL/HDL. Δ5 desaturase was inversely related to TG levels (Table 5). Δ6 and Δ9 desaturases have not been linked to any of the biochemistry related parameters assessed. Δ5 desaturase was directly related with arachidonic and docosahexaenoic and inversely with LA and DGLA (Table 5).

The linoleic acid was directly associated with the LA:DGLA ratio and inversely linked to all desaturases. The relations among FAs and projected desaturase activities were not affected by plasma Zn concentrations upon addition to the partial correlation models (data not presented).

### 3.6. Dietary Zn Intake of Participants

**Figure 1:**
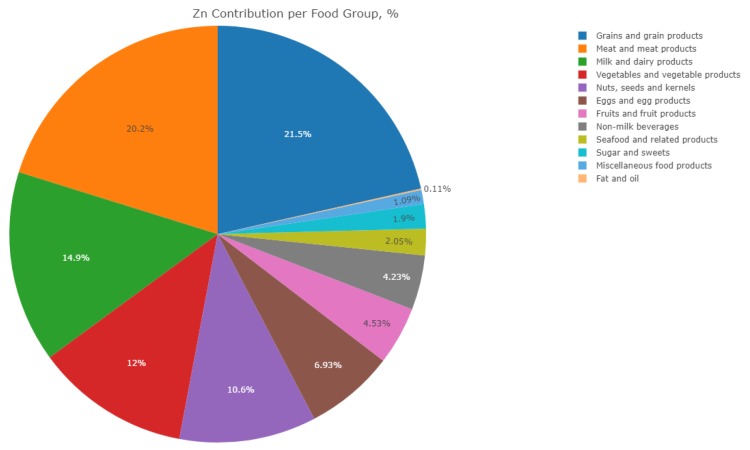
Top ten food sources of Zn in participants’ diets.

The average daily dietary Zn intake was 7.42 ± 1.82 mg/day with about 65% of study participants having intakes below the recommendations (8–11 mg/day) [55]. Grains and grain products (white and wholemeal bread) were the most significant sources of dietary Zn, contributing 21.5% to the total intake. Other important sources were meat and meat products (chicken meat) and milk and dairy products (yoghurt), with an estimated contribution of 20% and 15% to Zn intake, respectively (Table 6). Top ten food sources of Zn in participants’ diets are presented in Figure 1.

There was no correlation between the dietary Zn intake and plasma Zn status (*p* > 0.05). The dietary Zn intake was inversely correlated with the LA:DGLA ratio (r = −0.38, *p* = 0.05), while there was no significant relationship seen between the LA:DGLA ratio and dietary intake of Cu (r = −0.22, *p* = 0.26). Dietary Zn intake did not correlate with any of the biochemical or anthropometrical parameters measured within this study (data not presented). No associations were found between the dietary Zn intake and plasma status of individual FAs, except for the DGLA which was directly linked to the dietary Zn intake (r = 0.47, *p* = 0.03).

The average daily dietary intakes of Fe and Cu were 9.6 mg and 1.4 mg per day, respectively. Dietary intake of Cu was inversely linked to delta 5 desaturase (AA:DGLA), r = −0.395, *p* = 0.04. Dietary intake of individual fatty acids has mainly not been correlated with their status. The exceptions were 20:3 *n*6 and 20:5 *n*-3. The link between the eicosapentaenoic acid (EPA; C20:5 *n*-3) status and intake was r = 0.384, *p* = 0.048. Intake of DGLA (20:3 *n*6) has been linked to the status of EPA, DPA, DHA inversely (r = −0.41, r = −0.21, r = 0.47; *p* < 0.05, respectively) and with adrenic acid (C22:4 *n*6) directly (r = 0.439, *p* = 0.03).

## 4. Discussion

This study reports on the trace elements levels in plasma (Zn, Cu and Fe), anthropometrical, biochemical and fatty acid status in a sample of dyslipidemic adult subjects residing in Serbia. It examines the link between dietary Zn intake and concentrations of minerals with lipid profile indicators, fatty acid composition, and desaturase enzyme activities. People with dyslipidemia have inadequate dietary intakes of Zn, low plasma Zn status, and an altered FA composition. The concentrations of plasma Zn were linked to BMI and obesity, and there was no correlation between the dietary Zn intake and plasma Zn status. The ability of the LA:DGLA ratio to respond to variations in dietary Zn intake is reconfirmed. Cu, along with Zn, may have a role in activity of desaturase enzymes. Mean plasma Zn concentration was 0.75mg/L. The percentage of people with low plasma Zn concentrations using the IZiNGs criteria was 30% (50% with levels below 0.75mg/L), which indicates the presence of Zn deficiency in this study population [53]. There were no subjects with plasma Zn levels under 0.5mg/L, most likely due to a proficient homeostatic regulation of Zn. Our findings are in line with Obeid et al. [56] who also demonstrated a risk of Zn deficiency in Lebanese subjects with metabolic syndrome. The low plasma Zn concentrations were also seen in obese individuals, those with hypertension and type 2 diabetes [57,58]. Fe and Cu status data were within the estimated reference ranges for adult population, and similar to the values reported elsewhere [33,59]. The highest concentrations of Cu were seen in people with the lowest levels of plasma Zn. At the same time, the lowest concentrations of plasma Fe were found in a group of people with the lowest plasma Zn status. Decrease in serum Zn levels with a parallel increase in serum Cu concentrations has previously been reported in patients with myocardial infraction [60] and metabolic syndrome [61]. Similarly, concomitant decreases in the concentrations of both Fe and Zn have been described in the past [62,63]. No intercorrelations were seen between the concentrations of trace elements, which was also in accordance with some previously reported data [64,65]. Serum Cu:Zn is a useful indicator for identification of various diseases [66,67], including Zn deficiency [68]. Imbalances in the metabolism of Zn and Cu predispose to dyslipidemia, increased arteriosclerosis and cardiovascular diseases [69,70]. The ratio of Cu:Zn is believed to be clinically more relevant than the individual concentrations of either of these trace elements, and it is often used to show the general health state of an individual as it is very easily affected by inflammatory conditions [71]. The optimal plasma Cu:Zn ratio is in the range 0.7–1.00 [72] and the levels of 1.3 measured in this study certainly reflect a decreased nutritional Zn status, but also an inflammatory response. The reduction in plasma Zn levels in this study group could be explained by an increased uptake of Zn by tissues. Additionally, due to the presence of slight inflammation there may be change in the protein Zn-binding capacities [73]. Furthermore, the plasma Zn status did not correlate with dietary Zn intake, which is expected [33,74]. Sixty-five percent of our study sample did not meet dietary Zn requirements, which certainly contributed to low plasma Zn concentrations. Low dietary intake of Zn is found in people with a higher prevalence of coronary risk factors [70]. The bioavailability of Zn from the diet is highly dependent on the phytate: Zn molar ratio, and ratios above 15 are associated with a marked reduction in Zn absorption [53,75]. Dietary data are showing that major sources of Zn in our study group are plant sources (cereals-white and wholemeal bread), legumes and vegetables) with acknowledged high phytate content [76].

As majority of other databases, our database did not contain data on the amount of phytates in selected foods, so the exact phytate: Zn molar ratio of consumed foods was not obtained. However, when method by Jati et al. [77] was employed, Zn bioavailability from the diets consumed by our participants fitted into the group of ‘high bioavailability’ where ≤ 50% of total energy intake is accounted for from rice, other grains, other starchy staples, and pulses and nuts (35% in this group) and >5% (7.5% in our study sample) of total energy intake is accounted for by protein from fish, eggs, dairy, and meat. The association between anthropometric indicators and status of certain trace elements in a dyslipidemic population has been investigated in the past [78,79]. As with earlier findings [80,81] in our study no relationships were found between the statuses of trace elements with age. The increasing concentration of plasma Zn was associated with higher BMI and weight, similar to previous reports [33,82,83]. Yu et al. [61] reported comparable results in metabolic syndrome patients, higher serum Zn levels in those with higher weight. However, an inverse relation has also been reported [79,84], and future studies in large dyslipidemic cohorts are needed to derive decisive conclusions. Majority of our participants were either Zn deficient or with borderline Zn deficiency, which might be in line with potential mechanistic background of the observed correlations. Zn is an antioxidant, involved in the production of metalloenzymes and plays an important role in the metabolism of fats [85]. Zn dependent alpha2-glycoprotein (ZAG) is an adipokine which stimulates energy expenditure in skeletal muscle and brown adipose tissues resulting in reductions in body weight and TGs [86]. Additional studies with more diverse dietary and/or plasma Zn statuses of participants are needed to explore the causality of the associations observed herein. Similar to our study, previous reports failed to demonstrate the relationship between serum Cu and Fe concentrations with cholesterol and TG levels [17,82]. On the contrary, plasma Zn was directly and Cu:Zn ratio inversely associated with LDL/HDL, Cho/HDL and non-HDL/HDL. Inconsistent data are reported in the literature, a direct correlation between Zn and HDL [87,88] but also an inverse relationship [79,82]. An imbalance in the metabolism of Cu has been linked to hypercholesterolemia and an increased Cu:Zn superoxide dismutase was detected in obese individuals [89]. This obvious opposition between Zn and Cu has been acknowledged in hypertension [90], type 2 diabetes [91] and elevated blood pressure [92]. Zn can have an endogenous protective role against dyslipidemia and arteriosclerosis as it can inhibit the oxidation of LDL-C and protect against inflammatory diseases by inhibiting the activation of oxidative stress [82,93]. In our study, the insufficient dietary Zn intake and low plasma Zn concentrations could alter the lipid status and contribute to development of dyslipidemia. The wider ranging studies are now required to determine the exact order and underlying mechanisms of observed interactions further. Little is known about the relation between the desaturase enzyme activities and common dyslipidemic parameters. Increased Δ6 and Δ9 and reduced Δ5 desaturase activities are seen in people with chronic diseases [28,61,94]. In accordance with the data from other studies [29,95,96] herein the Δ6 and Δ9 desaturase activities were directly linked to BMI. Finally, the LA:DGLA ratio was inversely associated with BMI, Cho/HDL ratio and non-HDL/HDL and TG. The observed relations between the desaturases and biochemistry related parameters may result from the alterations in the desaturases encoding genes (FADS 1 and FADS2) that are linked to serum HDL-C and TG [97,98]. Moreover, desaturase enzymes are coupled to the NAD (P) H-cytochrome b5 electron transferrin chain and insufficient dietary Zn intake might affect the electron transferring chain and subsequently change the activities of desaturases and their relationship with lipid status parameters [99]. Furthermore, Zn modifies cyclooxy-genase activity and it acts as a co-enzyme for Δ6 desaturase [31,32,33,39]. The link between the Δ6 desaturase and Zn has been studied both in animals and humans [32,34,35,39]. The LA:DGLA ratio has been proposed as a potentially new biomarker of Zn status as it has been demonstrated that the ratio changes in accordance to dietary Zn manipulations [32,33,39]. However, the sensitivity of this biomarker to predict Zn status in Zn deficient human populations, as well as in people with an apparent presence of inflammatory conditions has not been explored so far. In this study, an inverse relationship among the LA:DGLA ratio and nutritional Zn status has been confirmed. Similarly, a noteworthy association was observed between the LA:DGLA ratio and plasma levels of Cu. Cu is the third most abundant essential trace element in the human body, and similarly to Zn, it also acts as a part of antioxidant enzymes that protects against free radicals [100]. Zn and Cu are known as interacting metallic elements, and an antagonistic relationship between Zn and Cu has been previously presented [89,101]. Herein, the lower than recommended dietary intake of Zn possibly contributed development of Zn deficiency and lead to a slightly misbalanced Cu:Zn ratio. The previously noted inverse trend between the LA:DGLA ratio and dietary Zn intakes has been reconfirmed in this study, and the detected borderline statistical significance was most likely due to the relatively uniform dietary Zn intakes of participants. Demonstrated altered Cu:Zn ratio demonstrates that the balance of these minerals is disturbed to a certain degree, in which situation Cu may be taking over Zn’s role, which is shown by a significant relationship seen between the plasma Cu concentrations and the LA:DGLA ratio. Cu may have a unique effect on long-chain FA metabolism [30]. In the early 1971, Brenner & Catala proposed that Cu can act on a metabolic pathway common to all FA desaturases, as a component of cupoprotein enzymes. As with findings of this study, a positive correlation has been described, an increase in desaturase activity with addition of Cu [102,103]. Active conversion of linoleic acid (LA) to its metabolites (GLA and DGLA) depends on the levels of these trace elements, higher levels of Zn and lower levels of Cu are an expected basis for an increased desaturase activity. It seems that Zn and Cu misbalance may be an active modifier of the LA:DGLA activity in dyslipidemia; however, this should be confirmed by further studies. The FA composition of serum phospholipids of this study group was similar to FA statuses of patients reported in other studies, but the values for *n*-3 PUFAs were lower from the values reported for healthy people [104,105,106]. The low intake and status of *n*-3 PUFA has been shown in obese populations and those with metabolic syndrome [61]. Low levels of *n*-3 PUFA are known to decrease the expression of mitochondrial messenger ribonucleic acid (RNA), which plays a role in a defense against obesity [107]. The overconsumption of *n*-6 PUFA and a high ratio of *n-*6 to *n*-3 PUFA may add to the increased obesity and dyslipidemia by stimulating low-grade chronic inflammation. *N*-3 PUFA influences obesity through eicosanoid modulation leading to a decrease in inflammation [106,108]. As expected there was the highest concentration of omega-6 FAs that are known as ‘pro-inflammatory’ and the lowest amount of omega-3 FA ‘anti-inflammatory’ in participants’ diets. The idea of *n*-6 FAs as ‘harmful’ is starting to change [109]. New evidence is showing that high levels of LA are inversely linked to the risk of development of cardiovascular diseases and mortality. The highest blood levels of LA in addition to *n*-3 FA reduced a risk of death for more than 50% [109]. However, the intake of *n*-3 FAs of participants in this group was below recommended intakes. In addition, the concentration of adrenic acid (22:4 *n*-6) increased in this study group, when compared to other reports in the literature [59,110]. 22:4 *n*-6 has a direct influence on the IKKb/NF-kB and inflammation signaling [111] which can promote type 2 diabetes and obesity [112], so adequate concentrations of this acid may be of great importance for prevention of obesity and dyslipidemia. Similarly, the *n*6/*n*3 PUFA ratio in Western diets (projected to be 10–25:1) has increased together with the prevalence of obesity and cardiovascular disease [113]. The reduction from 5:1 to 2:1 had a beneficial effect in preventing and treating a chronic disease [114]. In this study group, the ratio of 12:1 was higher than recommended, therefore achieving a much lower ratio may be beneficial for this dyslipidemic cohort. Very limited information is available on the role of Cu, Zn and Fe in FA metabolism in a dyslipidemic population. There were no statistically significant correlations between the plasma Zn and Fe with the statuses of any of the FAs measured within this study. Cu:Zn ratio was not linked to the status of individual FAs, while the plasma Cu was directly related to (alpha-linolenic acid (ALA,C18:3 *n*-3). Finally, it would be beneficial to explore whether the examined associations remain after the improved dietary intake of *n*-3 FAs and to what extend are potential dietary modifications able to diminish development of dyslipidemia and related disorders. Palmitoleic acid (C16:1 *n*-7), a monounsaturated FA correlated directly with BMI while oleic acid (C18:1 *n*-9) was linked to total cholesterol, which, if serum FA is used as a surrogate indicator of the dietary intake of FA, means that there is an increased dietary intake of monounsaturated FAs which contribute to increased cholesterol levels and obesity. This was supported by the dietary intake data, saturated fats contributed 13% to the total energy intake, while only 30% of fats consumed by this group was coming from PUFA sources. The direct correlation was also observed between the dietary intakes of fats with weight related indicators, which is in accordance with some previous reports [115,116]. Diets high in saturated fats and low in PUFAs may lead to the development of dyslipidemia. To sum up, Zn deficiency (and altered Cu:Zn ratio) could be important factors in decreased desaturase activity and modified FA metabolism. The obtained results should be interpreted with caution as reverse interconnections cannot be totally excluded. Although conducted within limited sample-size, our study results provide a valuable basis for further evaluation of the role of important trace elements (Fe, Cu and Zn) in the development of dyslipidemia and related conditions. The strengths of this study are the use of an entire range of independent biomarkers of dyslipidemic status: serum FAs, plasma mineral concentrations, anthropometrical, biochemical, and dietary intake data. Blood levels of several individual FAs are investigated rather than FA groups. Limitations of the present study include a lack of a directly measured inflammation indicators that could be assessed as potential confounders to relations of interest and a surrogate measure of desaturase enzyme activities. However, Cu:Zn ratio was used as an indicator for the presence of inflammation. Direct measurements of desaturase activities are challenging for ethical reasons, so the method used to estimate desaturase activity, the precursor-to-product ratio has frequently been used and has been shown to correlate well with desaturase expression in human liver biopsies [117]. Method employed to validate bioavailability of Zn is also generally accepted and often used in dietary data analysis. As far as we are aware, this is the first study that documents plasma levels of Zn, Cu, and Fe in relation to an entire range of anthropometrical, biochemical, FA status components, and desaturases activities in people suffering from dyslipidemia. It was certainly the first study of this type in Serbia.

## 5. Conclusions

The results offered in the present study add novel information to the limited body of literature on the relationship between the important trace elements (Zn, Cu and Fe) with fat intake and FA metabolism in people with dyslipidemia. We showed that people with dyslipidemia have inadequate dietary intakes of Zn and a low plasma Zn status. The increased Cu:Zn ratio indicates the presence of a low-grade inflammatory conditions in this group. There were no correlations between the plasma Zn and dietary Zn intake, and the increase in the concentration of plasma Zn was correlated with obesity. Observed alterations in serum FA composition are most likely caused by Zn deficiency and an altered Cu:Zn ratio. The inverse correlation between the dietary Zn intakes and the LA:DGLA ratio has been reconfirmed. The LA:DGLA ratio was directly linked to plasma levels of Cu, which implies that Cu, in addition to Zn, may have a role in activity of desaturase enzymes, particularly in Zn deficient people and in those affected by inflammatory conditions. Dietary modifications related to the microelements appear as suitable therapeutic strategies for people suffering from dyslipidemia, and further research in the field is needed.

## Figures and Tables

**Table 1:** Baseline characteristics of study participants.

	Whole Sample (*n* = 27)	1st Zn Tertile (*n* = 8)	2nd Zn Tertile (*n* = 7)	3rd Zn Tertile (*n* = 8)	*p*-Value
**Clinical Parameters**					
Age, Years	57.00 [16.00]	59.50 [15.00]	63.00 [24.00]	53.00 [14.00]	*p* = 0.781
Weight, Kg	77.50 [31.30]	68.65 [18.37]	76.4 [22.70]	95.90 [21.10]	***p* = 0.001**
BMI, Kg/M^2^	27.10 [8.30]	24.85 [8.97]	26.60 [3.80]	34.10 [9.82]	***p* = 0.009**
Plasma Zn, Mg/L	0.75 [0.14]	0.64 [0.09]	0.75 [0.05]	0.86 [0.08]	***p* < 0.001**
Plasma Fe, Mg/L	0.85 [0.65]	0.82 [0.23]	1.10 [0.91]	0.90 [0.76]	*p* = 0.328
Plasma Cu, Mg/L	0.98 [0.16]	1.01 [0.06]	0.94 [0.11]	0.95 [0.18]	*p* = 0.289
Cu:Zn	1.27 [0.39]	1.60 [0.36]	1.26 [0.10]	1.05 [0.31]	***p* < 0.001**
Total Cholesterol, Mmol/L	5.87 [1.26]	5.87 [0.67]	6.65 [1.29]	5.95 [0.96]	*p* = 0.383
HDL-C, Mmol/L	1.41 [0.64]	1.69 [0.35]	1.28 [0.60]	1.22 [0.59]	*p* = 0.318
LDL-C, Mmol/L	3.95 [0.79]	3.59 [0.60]	4.33 [1.00]	3.83 [0.58]	*p* = 0.076
TG, Mmol/L	1.20 [0.69]	1.00 [0.33]	1.56 [0.49]	1.37 [0.77]	*p* = 0.928
FPG, Nmol/L	5.17 [0.98]	4.96 [0.57]	5.76 [1.11]	5.53 [1.16]	*p* = 0.166
Systolic BP, MmHg	131.00 [32.00]	137.00 [51.75]	120.00 [29.00]	136.50 [13.00]	*p* = 0.295
Diastolic BP, MmHg	81.00 [22.00]	76.50 [17.75]	70.00 [19.00]	90.50 [14.50]	*p* = 0.075
**Fatty Acids in Plasma Phospholipids%**					
18:1 *n*-9	8.11 [1.89]	8.04 [1.27]	8.12 [2.40]	8.02 [1.59]	*p* = 0.951
18:2 *n*-6	24.01 [5.09]	26.62 [5.51]	25.69 [6.79]	21.99 [4.10]	*p* = 0.385
18:3 *n*-3	0.08 [0.068]	0.11 [0.05]	0.07 [0.05]	0.08 [0.08]	*p* = 0.214
20:3 *n*-6	2.83 [1.28]	2.71 [1.17]	2.83 [1.29]	2.71 [1.32]	*p* = 0.958
20:4 *n*-6	11.67 [2.84]	10.86 [4.90]	11.58 [2.72]	12.27 [1.03]	*p* = 0.330
20:5 *n*-3	0.27 [0.21]	0.27 [0.57]	0.23 [0.09]	0.42 [0.16]	*p* = 0.239
22:4 *n*-6	0.36 [0.17]	0.33 [0.22]	0.40 [0.16]	0.37 [0.15]	*p* = 0.775
22:5 *n*-3	0.55 [0.15]	0.27 [0.57]	0.50 [0.16]	0.56 [0.19]	*p* = 0.436
22:6 *n*-3	2.49 [1.42]	2.44 [1.95]	2.40 [0.17]	3.32 [1.33]	*p* = 0.403
Δ5-Desaturase	3.68 [1.80]	3.47 [0.61]	3.82 [1.53]	4.52 [2.88]	*p* = 0.406
Δ6-Desaturase	0.009 [0.003]	0.007 [0.005]	0.009 [0.003]	0.009 [0.003]	*p* = 0.866
LA: DGLA	8.81 [3.85]	9.45 [6.18]	8.93 [3.94]	8.47 [2.94]	*p* = 0.856
Δ9-Desaturase	0.14 [0.01]	0.013 [0.004]	0.014 [0.010]	0.012 [0.005]	*p* = 0.145

Data are presented as median [interquartile range] in complete sample and according to the tertiles of plasma zinc levels. Bolded *p*-values represent the results of one-way ANOVA or Kruskal Wallis test on significant differences between the Zn tertiles. Abbreviations: BMI—body mass index; HDL-C—high-density lipoprotein cholesterol; LDL-C—low-density lipoprotein cholesterol; Body Fat%-body fat percentage; TG–triglycerides; FPG-fasting plasma glucose; BP-blood pressure; LA: DGLA-linolenic acid/dihomo-gama-linolenic acid ratio.

**Table 2:** Correlations between plasma levels of trace elements (Zn, Fe, Cu) and Cu:Zn ratio with anthropometrical and biochemical parameters in study subjects.

	BMI	Body Fat%	FPG	TG	Total Cholesterol	LDL-C	HDL-C	Cho/HDL	LDL/HDL	Non-HDL/HDL
**Cu**	r = −0.147 *p* = 0.504 504504	r = 0.177 *p* = 0.417	r = −0.156 *p* = 0.477	r = −0.161 *p* = 0.463	r = 0.040 *p* = 0.856	r = −0.006 *p* = 0.978	r = 0.346 *p* = 0.106	r = −0.335 *p* = 0.118	r = −0.269 *p* = 0.215	r = −0.335 *p* = 0.118
**Fe**	r = −0.161 *p* = 0.463	r = −0.497 *p* = 0.016	r = −0.097 *p* = 0.660	r = 0.128 *p* = 0.562	r = −0.198 *p* = 0.364	r = −0.116 *p* = 0.943	r = −0.272 *p* = 0.210	r = 0.195 *p* = 0.373	r = 0.212 *p* = 0.331	r = 0.195 *p* = 0.373
**Zn**	r = 0.536 *p* = 0.021	r = 0.161 *p* = 0.798	r = 0.386 *p* = 0.069	r = 0.269 *p* = 0.215	r = 0.192 *p* = 0.379	r = 0.289 *p* = 0.181	r = −0.307 *p* = 0.155	r = 0.469 *p* = 0.024	r = 0.499 *p* = 0.015	r = 0.469 *p* = 0.024
**Cu:Zn**	r = −0.488 *p* = 0.018	r = −0.065 *p* = 0.769	r = −0.349 *p* = 0.102	r = −0.298 *p* = 0.167	r = −0.109 *p* = 0.622	r = −0.203 *p* = 0.354	r = 0.430 *p* = 0.041	r = −0.541 *p* = 0.008	r = −0.518 *p* = 0.011	r = −0.542 *p* = 0.008

Abbreviations: Body Fat%-body fat percentage; FPG-fasting plasma glucose; TG–triglycerides; Cho/HDL–total cholesterol/HDL-C ratio.

**Table 3:** Correlations of plasma concentrations of trace elements (Zn, Fe, Cu) and Cu:Zn ratio with plasma fatty acids and estimated desaturase activity in study subjects.

	18:1 *n*-9	18:2 *n*-6	18:3 *n*-3	20:3 *n*-6	20:4 *n*-6	20:5 *n*-3	22:4 *n-*6	22:5 *n*-3	22:6 *n*-3	LA: DGLA	Δ5-Desaturase	Δ6-Desaturase
**Cu**	r = 0.433 *p* = 0.039	r = 0.265 *p* = 0.222	r = 0.542 *p* < 0.001	r = −0.194 *p* = 0.060	r = −0.267 *p* = 0.217	r = 0.231 *p* = 0.290	r = −0.357 *p* = 0.095	r = −0.108 *p* = 0.624	r = 0.162 *p* = 0.461	r = 0.293 *p* = 0.175	r = −0.010 *p* = 0.963	r = −0.196 *p* = 0.370
**Fe**	r = 0.415 *p* = 0.049	r = 0.059 *p* = 0.788	r = −0.503 *p* = 0.014	r = 0.157 *p* = 0.474	r = −0.092 *p* = 0.677	r = −0.021 *p* = 0.925	r = 0.113 *p* = 0.609	r = 0.045 *p* = 0.837	r = −0.049 *p* = 0.823	r = −0.092 *p* = 0.677	r = −0.145 *p* = 0.508	r = 0.137 *p* = 0.532
**Zn**	r = 0.118 *p* = 0.330	r = −0.182 *p* = 0.380	r = −0.152 *p* = 0.696	r = 0.007 *p* = 0.975	r = 0.118 *p* = 0.391	r = 0.131 *p* = 0.553	r = 0.108 *p* = 0.622	r = 0.077 *p* = 0.728	r = 0.214 *p* = 0.327	r = −0.132 *p* = 0.058	r = 0.168 *p* = 0.471	r = 0.168 *p* = 0.443
**Cu:Zn**	r = −0.130 *p* = 0.517	r = 0.297 *p* = 0.168	r = 0.449 *p* = 0.026	r = −0.118 *p* = 0.593	r = −0.305 *p* = 0.157	r = 0.051 *p* = 0.816	r = −0.345 *p* = 0.107	r = −0.111 *p* = 0.615	r = −0.036 *p* = 0.872	r = −0.243 *p* = 0.244	r = −0.120 *p* = 0.586	r = −0.243 *p* = 0.264

Abbreviations: LA: DGLA-linolenic acid/dihomo-gama-linolenic acid ratio.

**Table 4:** Correlations between individual plasma fatty acids with anthropometrical and biochemical parameters in study subjects.

	BMI	Body Fat%	TG	Cholesterol	LDL-C	HDL-C
**18:1 *n*-9**	r = −0.065 *p* = 0.746	r = 0.193 *p* = 0.334	r = −0.330 *p* = 0.093	r = 0.474 *p* = 0.012	r = 0.266 *p* = 0.180	r = 0.660 *p* < 0.001
**18:2 *n*-6**	r = −0.458 *p* = 0.016	r = −0.317 *p* = 0.107	r = −0.028 *p* = 0.888	r = −0.362 *p* = 0.063	r = −0.310 *p* = 0.116	r = −0.107 *p* = 0.597
**18:3 *n*-3**	r = −0.057 *p* = 0.777	r = 0.301 *p* = 0.127	r = −0.127 *p* = 0.527	r = 0.005 *p* = 0.979	r = −0.181 *p* = 0.367	r = 0.233 *p* = 0.243
**20:3 *n*-6**	r = 0.270 *p* = 0.173	r = 0.168 *p* = 0.403	r = 0.560 *p* = 0.002	r = 0.185 *p* = 0.355	r = 0.243 *p* = 0.222	r = −0.339 *p* = 0.083
**20:4 *n-*6**	r = 0.416 *p* = 0.031	r = 0.245 *p* = 0.217	r = −0.135 *p* = 0.502	r = 0.118 *p* = 0.557	r = 0.089 *p* = 0.658	r = 0.109 *p* = 0.614
**20:5 *n*-3**	r = 0.223 *p* > 0.05	r = 0.261 *p* > 0.05	r = −0.106 *p* > 0.05	r = 0.029 *p* > 0.05	r = −0.016 *p* > 0.05	r = 0.134 *p* > 0.05
**22:4 *n*-6**	r = −0.080 *p* = 0.970	r = −0.204 *p* = 0.306	r = 0.102 *p* = 0.612	r = −0.036 *p* = 0.860	r = −0.168 *p* = 0.967	r = −0.162 *p* = 0.421
**22:5 *n*-3**	r = 0.009 *p* = 0.965	r = 0.011 *p* = 0.957	r = −0.107 *p* = 0.597	r = 0.011 *p* = 0.955	r = −0.028 *p* = 0.692	r = 0.080 *p* = 0.889
**22:6 *n*-3**	r = 0.294 *p* = 0.136	r = 0.201 *p* = 0.314	r = 0.024 *p* = 0.905	r = 0.029 *p* = 0.885	r = 0.153 *p* = 0.447	r = −0.143 *p* = 0.477

Abbreviations: Body Fat%-body fat percentage; TG–triglycerides.

**Table 5:** Correlations between LA:DGLA ratio and desaturase activities with the status of individual plasma fatty acids, BMI, and biochemical parameters in study subjects.

	18:2 *n*-6	20:3 *n*-6	20:4 *n*-6	22:6 *n*-3	BMI	TG	Cho/HDL	LDL/HDL	Non-HDL/HDL
**LA:DGLA Ratio**	r = 0.470 *p* = 0.013	r = −0.889 *p* < 0.001	r = −0.209 *p* = 0.296	r = −0.008 *p* = 0.969	r = −0.418 *p* = 0.030	r = −0.506 *p* = 0.007	r = −0.433 *p* = 0.024	r = −0.362 *p* = 0.063	r = −0.433 *p* = 0.024
**Δ5-Desaturase**	r = −0.406 *p* = 0.035	r = −0.710 *p* < 0.001	r = 0.694 *p* < 0.001	r = 0.441 *p* = 0.021	r = 0.085 *p* = 0.672	r = −0.392 *p* = 0.043	r = −0.301 *p* = 0.243	r = −0.240 *p* = 0.454	r = −0.301 *p* = 240
**Δ 6-Desaturase**	r = −0.627 *p* < 0.001	r = 0.200 *p* = 0.317	r = 0.311 *p* = 0.114	r = −0.075 *p* = 0.710	r = 0.398 *p* = 0.040	r = 0.194 *p* = 0.334	r = 0.125 *p* = 0.533	r = 0.132 *p* = 0.510	r = 0.127 *p* = 0.527
**Δ 9-Desaturase**	r = −0.402 *p* = 0.037	r = 0.212 *p* = 0.289	r = 0.056 *p* = 0.781	r = −0.086 *p* = 0.669	r = 0.284 *p* = 0.152	r = 0.239 *p* = 0.230	r = 0.112 *p* = 0.578	r = 0.091 *p* = 0.653	r = 0.111 *p* = 0.581

Abbreviations: TG–triglycerides; Cho/HDL–total cholesterol/HDL-C ratio.

**Table 6:** Contribution of different food groups to dietary Zn intake of study participants.

Food Group	Average Zn Intake, Mg	Contribution to Zn Intake, %
Grains and Grain Products	1.595	21.49
Meat and Meat Products	1.499	20.20
Milk and Dairy Products	1.104	14.88
Vegetables and Vegetable Products	0.888	11.97
Nuts, Seeds, and Kernels	0.789	10.63
Eggs and Egg Products	0.514	6.93
Fruit and Fruit Products	0.336	4.53
Non-Milk Beverages	0.314	4.23
Sugar and Sweets	0.141	1.90
Seafood and Related Products	0.152	2.05
Miscellaneous Food Products	0.081	1.09
Fat and Oil	0.008	0.11
